# Antiprotozoal and Antibacterial Activity of Ravenelin, a Xanthone Isolated from the Endophytic Fungus *Exserohilum rostratum*

**DOI:** 10.3390/molecules26113339

**Published:** 2021-06-02

**Authors:** Jeferson Rodrigo Souza Pina, João Victor Silva-Silva, Josiwander Miranda Carvalho, Heriberto Rodrigues Bitencourt, Luciano Almeida Watanabe, Juan Matheus Pereira Fernandes, Guilherme Eduardo de Souza, Anna Caroline Campos Aguiar, Rafael Victorio Carvalho Guido, Fernando Almeida-Souza, Kátia da Silva Calabrese, Patrícia Santana Barbosa Marinho, Andrey Moacir do Rosario Marinho

**Affiliations:** 1 Post-Graduate Program in Chemistry, Federal University of Pará, 66075-110 Belém, Brazil; konanquim@gmail.com (J.R.S.P.); mcwander@hotmail.com (J.M.C.); heriberto.ufpa@gmail.com (H.R.B.); lucianowat@yahoo.com.br (L.A.W.); pat@ufpa.br (P.S.B.M.); 2 Laboratory of Immunomodulation and Protozoology, Oswaldo Cruz Institute, Oswaldo Cruz Foundation, 21040-360 Rio de Janeiro, Brazil; jvssilva89@gmail.com (J.V.S.-S.); juanfernandes222@gmail.com (J.M.P.F.); fernandoalsouza@gmail.com (F.A.-S.); kscalabrese@gmail.com (K.d.S.C.); 3 São Carlos Institute of Physics, University of São Paulo, São Carlos, 13566-590 São Paulo, Brazil; guilherme.eduardo.souza@usp.br (G.E.d.S.); carolcaguiar@yahoo.com.br (A.C.C.A.); rvcguido@ifsc.usp.br (R.V.C.G.); 4 Post-Graduate Program Animal Sciences, State University of Maranhão, 65055-310 São Luís, Brazil

**Keywords:** antimicrobial, antiprotozoan, polyketides, fungi, xanthone

## Abstract

The natural compound ravenelin was isolated from the biomass extracts of *Exserohilum rostratum* fungus, and its antimicrobial, antiplasmodial, and trypanocidal activities were evaluated. Ravenelin was isolated by column chromatography and HPLC and identified by NMR and MS. The susceptibility of Gram-positive and Gram-negative bacteria strains to ravenelin was determined by microbroth dilution assay. Cytotoxicity was evaluated in hepatocarcinoma cells (HepG2) and BALB/c peritoneal macrophages by using MTT. SYBR Green I-based assay was used in the asexual stages of *Plasmodium falciparum*. Trypanocidal activity was tested against the epimastigote and intracellular amastigote forms of *Trypanosoma cruzi*. Ravenelin was active against Gram-positive bacteria strains, with emphasis on *Bacillus subtilis* (MIC value of 7.5 µM). Ravenelin’s antiparasitic activities were assessed against both the epimastigote (IC_50_ value of 5 ± 1 µM) and the intracellular amastigote forms of *T. cruzi* (IC_50_ value of 9 ± 2 µM), as well as against *P. falciparum* (IC_50_ value of 3.4 ± 0.4 µM). Ravenelin showed low cytotoxic effects on both HepG2 (CC_50_ > 50 µM) and peritoneal macrophage (CC_50_ = 185 ± 1 µM) cells with attractive selectivity for the parasites (SI values > 15). These findings indicate that ravenelin is a natural compound with both antibacterial and antiparasitic activities, and considerable selectivity indexes. Therefore, ravenelin is an attractive candidate for hit-to-lead development.

## 1. Introduction

Secondary metabolites produced by microorganisms, including fungi and bacteria, have shown useful applications in different areas of human life development [1]. Penicillin is the most well-known example of a secondary metabolite produced by a microorganism that has been used against microbial diseases [2].

Fungi produce diverse classes of bioactive compounds [3,4]. Several studies have demonstrated the potential of endophytic fungi as producers of bioactive compounds. The natural products of endophytic fungi show a variety of both biological activities and chemical classes, including alkaloids, steroids, terpenoids, flavonoids, glycosides, xanthones, isocoumarins, quinones, phenylpropanoids, lignans, aliphatic metabolites, and lactones, among others [5].

The increasing number of resistant pathogens (bacteria and fungi) to the antimicrobials available [6] and the loss of effectiveness to antiprotozoal treatment [7] have motivated the investigation of bioactive compounds from natural sources. In this sense, secondary metabolites isolated from endophytic fungi may play a role against microbial and parasite resistance [8]. For instance, isocoumarins analogs have been obtained from cultures of *E. rostratum* isolated as endophytic fungi of *Stemona sp.*, and had their biological activity assessed against a resistant strain of *P. falciparum* (K1, multidrug-resistant strain). The most potent compound of the series showed inhibitory activity in the submicromolar range (IC_50_ of 0.68 µM) [9].

Xanthones are natural products isolated from plants and microorganisms, including endophytic fungi [10,11]. Despite its simple chemical structure, the investigations around the xanthone core have generated a large number of xanthone analogs [12]. These analogs have shown a variety of biological activities [13], including antidepressant and anxiolytic [14], antitubercular [15], antimicrobial [16], anticancer [17], antiviral [18], antioxidant [19], anti-inflammatory [20], and antiparasitic [21].

In this work, the fungus *E. rostratum* was isolated from *Phanera splendens* (Kunth) Vaz (Leguminosae), an endemic medicinal plant of the Amazon region known as “Tortoise Ladder” and used in folk medicine against infectious, inflammatory, and diabetes processes [22], was studied. There are few studies in the literature on the chemistry of the genus *Exserohilum*. A previous chemical study on *E. rostratum* reported the isolation of polyketides with lactone skeleton of tri-substituted α-pyrone with moderate antimicrobial activity [23]. Then, xanthone ravenelin was obtained by us, and the aim of this work was to study the therapeutic potential of ravenelin as antimalarial, antichagasic or antileishmanial. Therefore, to the best of our knowledge, this is the first report about its antiprotozoal profile.

## 2. Results

### 2.1. Isolation and Characterization of Ravenelin

**Figure 1:**
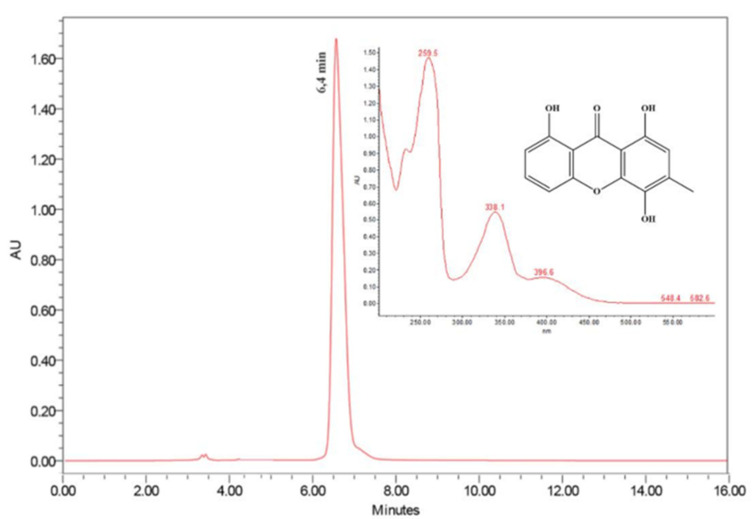
Preparative HPLC-PAD chromatogram at 254 nm of fraction A3 obtained of ethyl acetate from *E. rostratum.* UV-Vis spectrum of ravenelin (t_R_ = 6.4 min). Sunfire™ prep C18 OBD column (5 µm, 19 mm × 150 mm), 500 μL of injected volume of the sample, gradient elution H_2_O/MeOH (90–100%) for 16 min, flow 9.0 mL/min, PAD range 210–600 nm.

The compound ravenelin **1** was isolated from the fraction A3 of the ethyl acetate extract from *E. rostratum* by a preparative high-performance liquid chromatography with a photodiode array detector (HPLC-PAD) (Figure 1). The electrospray ionization mass spectrum (ESIMS) (-) of compound **1** showed *m*/*z* 257.2 [M-H]^−^, which, combined with the NMR data, allowed us to propose the molecular formula C_14_H_10_O_5_. The structure of the isolated compound was determined by one- (1D) and two-dimensional (2D) NMR, FTIR, and MS data (Supplementary Material).

### 2.2. Antibacterial Activity of Ravenelin

The antibacterial activities of ravenelin were evaluated against Gram-positive and Gram-negative bacteria (Table 1). The results indicated that ravenelin did not show inhibitory activity against Gram-negative strains (MIC > 1000 μM), but showed inhibition against *Staphylococcus aureus* (MIC value of 484 μM or 125 µg/mL) and *Bacillus subtilis* (MIC value of 7.5 μM or 1.95 µg/mL). Amoxicillin and terramycin were used as positive controls (Table 1).

### 2.3. Antiplasmodial and Anti-Trypanosoma Effect of Ravenelin

Ravenelin was tested in vitro against cultures of *P. falciparum* 3D7, a chloroquine-sensitive strain, and *T. cruzi* (epimastigote and intracellular amastigote forms) (Table 2). Artesunate and benznidazole were used as positive controls for inhibition against *P. falciparum* and *T. cruzi*, respectively. The compound showed antiplasmodial (IC_50_ = 3.4 μM) and trypanocidal activities (IC_50_^Epi^ = 5 μM and IC_50_^ama^ = 9 μM) in the low micromolar range.

**Figure 2:**
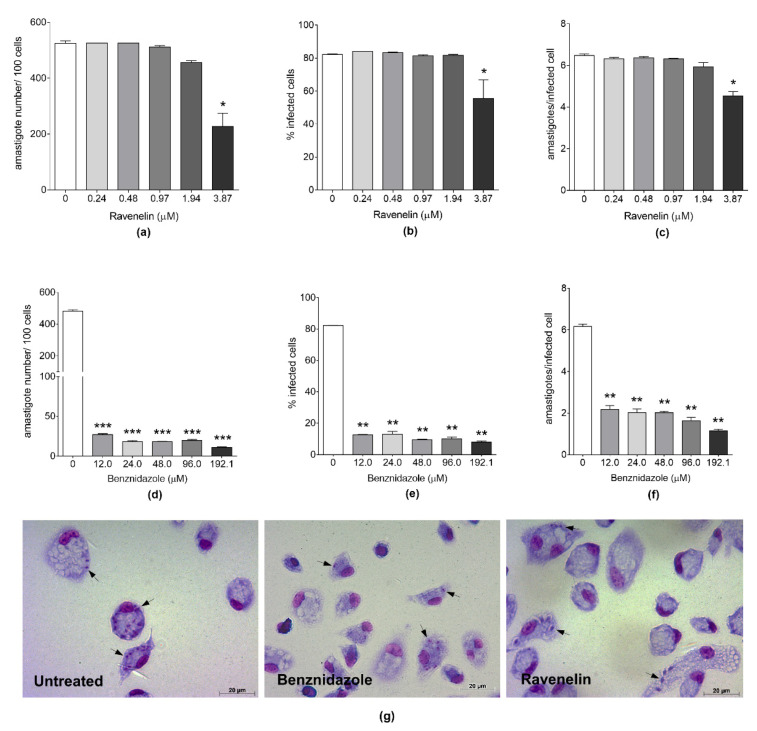
Infection parameters of BALB/c peritoneal macrophages infected with *T. cruzi* and treated for 24 h with ravenelin or benznidazole. (**a**–**f**) Parameters of infection and (**g**) light microscopy after ravenelin or benznidazole treatment at 3.87 μM or 192.1 μM, respectively. Intracellular amastigotes inside macrophages (black arrows). Data represent mean ± standard deviation of two independent experiments conducted in triplicate. * *p* < 0.05, ** *p *< 0.01 and *** *p *< 0.001, when compared to the untreated group by the Mann–Whitney test.

The analysis of the infection indicated that ravenelin determined a statistically significant reduction in the number of amastigotes per 100 cells (*p* = 0.0226; Figure 2a), the percentage of infected cells (*p* = 0.0286; Figure 2b), and the mean of amastigotes per infected cell (*p* = 0.0119; Figure 2c), but only at the greatest concentration tested (3.87 μM or 1 μg/mL). By contrast, benznidazole showed a significant reduction in all parameters of infection at all concentrations tested (Figure 2d–f). The alterations in intracellular amastigote form of *T. cruzi* after treatment with ravenelin are represented in photomicrography images of Figure 2g.

### 2.4. Cytotoxicity and Selectivity Index of Ravenelin

The cytotoxic effect and analysis of the selectivity index (SI = CC_50_/IC_50_) indicate whether a compound is selectively toxic to the parasite compared to other cells. In this sense, ravenelin was selective for *P. falciparum* (CC_50_ > 50; SI > 15) in comparison with HepG2 cells (Table 3). Similarly, the cytotoxicity analysis of peritoneal macrophages treated with ravenelin showed a CC_50_ value of 185 µM (47.7 µg/mL) and a selectivity index of 21 (Table 3).

## 3. Discussion

Ravenelin was isolated by chromatographic procedures and identified by 1D and 2D NMR, FTIR, and MS data. NMR data of ravenelin reported herein were compared with those published in the literature for the xanthone ravenelin [24,25].

In this study, ravenelin was tested for antibacterial activities against *E. coli*, *P.* *aeruginosa*, *B. subtilis*, *S. Typhimurium*, and *S. aureus*. In general, ravenelin exhibited pronounced antibacterial activity against the Gram-positive bacteria (*B. subtilis* and *S. aureus*) only. The tetraoxygenated xanthone derivatives isolated from immature fruits of *Garcinia cowa* also showed antibacterial activities against the Gram-positive strains, especially against *B. subtilis* (MIC value of 0.25–4 µg/mL) [26]. In another study, α-mangostin, the major xanthone derivative from *Garcinia mangostana*, was investigated for antimicrobial activity [27]. The natural product was a poor inhibitor of *E. coli* and *P. aeruginosa* (IC_50_ > 200 µg/mL). However, the molecule showed inhibitory activity against both *B. subtilis* and *S. aureus* (MIC 1.6 and 3.2 µg/mL) [27].

The investigation of the biological activities of secondary metabolites produced by associated fungi such as *Talaromyces funiculosus* and *Diorygma hieroglyphicum* led to the isolation of ravenelin, which exhibited an MIC value of 372 µM against *S. aureus* [28]. These data, together with antibacterial data obtained by Padhi et al. (2019), which assessed the activity of ravenelin, suggest a possible affinity of this xanthone for Gram-positive bacteria.

There are few studies addressing the antiprotozoal activity of xanthones isolated from endophytic fungi. One of these studies demonstrated the antiplasmodial and trypanocidal activity for this chemical class. For instance, ascherxanthone A, isolated from the fungus *Aschersonia* sp., exhibited significant inhibitory activity against *P. falciparum* (K1 strains), with an IC_50_ value of 0.20 µg/mL. Nonetheless, the compound showed noticeable cytotoxicity to Vero cells (IC_50_ = 0.80 µg/mL) [29]. Phomoxanthones A and B, two xanthone dimers isolated from the endophytic fungus *Phomopsis* sp. BCC 1323, exhibited significant antiplasmodial activity against *P. falciparum* (K1 strain) with IC_50_ values of 0.11 and 0.33 µg/mL, respectively, and a moderate cytotoxic effect on Vero cells (IC_50_ 1.4 and 1.8 µg/mL, respectively) [30]. Similar results were observed with α-mangostin, a low micromolar *P. falciparum* inhibitor (IC_50_ = 2.2 µM) with cytotoxic effects on MRC-5 cells in the same activity range (IC_50_ = 7.5 µM), thereby suggesting non-specific inhibition. These results agree with the data obtained in this study, which indicated that ravenelin is active for *P. falciparum* with inhibitory activity at the low micromolar range. However, ravenelin showed low cytotoxic activity on HepG2 cells, suggesting the natural compound has an acceptable selectivity index (SI > 15) related to the known xanthone derivatives.

Moreover, ravenelin showed activity against both the epimatigote and the intracellular amastigote forms of *T. cruzi*. Xanthone derivatives with trypanocidal potential have also been reported. For example, Dua et al. [31] isolated four xanthones from the roots of *Andrographis paniculata* and tested the compounds against trypomastigote forms of *T. brucei brucei*, intracellular amastigotes of *T. cruzi*, and *Leishmania infantum*. The compound 1,2-dihydroxy-6,8-dimethoxy-xanthone showed promising activity against *T. b. brucei* and *L. infantum* with IC_50_ of 4.6 µg/mL and 8 µg/mL, respectively [31]. Similarly, Al-Massarani et al. [27] demonstrated that α-mangostin was active against intracellular amastigotes of *L. infantum*, and trypomastigotes of *T. brucei*, and *T. cruzi*, with IC_50_ values in the low micromolar range (IC_50_s between 8.0 and 9.0 µM). In addition, Dua et al. [31] indicated that 1,2-dihydroxy-6,8-dimethoxy-xanthone showed CC_50_ values > 32 μg/mL against mammalian cells (MRC-5, human lung fibroblast), as well as α-mangostin, showed cytotoxicity on MRC-5 cells (CC_50_ = 7.5 µM) [5,27]. Our study indicated that ravenelin showed cytotoxic effects on peritoneal macrophages at a concentration 3.6-fold lower than benznidazole (the reference drug). However, in comparison with trypanocidal activity, ravenelin showed substantial selectivity indexes of 37 and 21 against the epimastigote and intracellular amastigote forms of *T. cruzi*, respectively. Selectivity indexes greater than 10 indicate that biological efficacy is not due to in vitro cytotoxicity [32].

## 4. Materials and Methods

### 4.1. Plant Material

*Phanerasplendens* (Kunth) Vaz (Leguminosae) was collected in the city of Belém, Pará State, Brazil, in December 2016, and a voucher specimen (number IAN 177.179) was deposited at the Herbarium of the Brazilian Agricultural Research Corporation (EMBRAPA).

### 4.2. E. rostratum Isolation

The *E. rostratum* fungus was isolated from in natura healthy tissues of *P. splendens* (Kunth) Vaz (Leguminosae) through a sequence of immersions of small pieces of plant material in hexane, 4% aqueous solution of sodium hypochlorite, 70% ethanol, and sterile water. The plant material was inoculated into a 9 cm Petri dish containing a Potato Dextrose Agar (PDA) culture medium (HiMedia, Mumbai, India) and incubated for seven days at 25 °C for colony growth, which was purified by successive sampling. The *E. rostratum* fungus was identified by DNA sequencing at the Institute of Biological Sciences, Federal University of Pará (UFPA).

### 4.3. Culture of E. Rostratum in Rice and Compound Isolation

The fungus was cultivated in forty-five Erlenmeyer flasks (500 mL) containing 90 g of rice and 75 mL of distilled water per flask. Three flasks (rice only) were used as control. Small pieces of PDA from the Petri dish containing mycelium of *E. rostratum* were transferred under sterile conditions to forty-two Erlenmeyer flasks previously autoclaved for 45 min at 121 °C, and stored for twenty-eight days at 25 °C for colony growth. The biomass obtained was macerated with ethyl acetate (2L, three times). After simple filtration, the solution was concentrated in a rotary evaporator, and the ethyl acetate biomass extracts (22.5 g) were obtained. A part of the ethyl acetate extract (3.0 g) was fractionated on a chromatography column using silica gel SiliaSphere^TM^ (Silicycle, Québec, QC, Canada, 60–200 mesh) as stationary phase and hexane, ethyl acetate, and methanol as mobile phase in the polarity gradient, and A1 to A6 fractions were given (A1: Hexene; A2: Hexene/Ethyl Acetate 8:2; A3: Hexene/Ethyl Acetate 1:1; A4: Hexene/Ethyl Acetate 2:8; A5: Ethyl Acetate; and A6: Methanol, 1L each). Afterwards, the A1 to A6 solution fractions were concentrated in the rotatory evaporator (Quimis, Diadema, SP, Brazil) and were analyzed by thin-layer chromatography (TLC) on silica gel and mobile phase hexane/ethyl acetate 6:4. The compound ravenelin (25 mg) was isolated from fraction A3 by high-performance liquid chromatography HPLC-PAD in preparative mode using a Waters 1525 Binary HPLC Pump (Waters, Milford, MA, USA) equipped with Waters 2998 photodiode array detector and Sunfire™ prep C18 column (5 µm, 19 mm × 150 mm). Chromatographic separation occurred with 500 μL of the injected volume of the sample with elution of a gradient of H_2_O/MeOH (90–100%) for 16 min, with a flow rate of 9.0 mL/min. The wavelength was scanned in the range of 210–600 nm. The wavelength monitored was 254 nm.

### 4.4. NMR and MS Analysis

The mass spectrum was obtained in negative ion mode using an Acquity tandem quadrupole detector (TQD) (Waters, Milford, MA, USA) mass spectrometer equipped with an electrospray ionization source (ESI). The 1D and 2D NMR spectra were recorded on a Bruker Ascend 400 (Bruker, Fällanden, Switzerland). Ravenelin was solubilized in acetone-d_6_ to record NMR spectra. The chemical shifts are given in delta (δ) values and the coupling constants (*J*) in Hertz (Hz), and the solvent signal (acetone-d_6_) was used as reference. The IR spectrum was obtained on an Agilent Cary 630 FTIR (Agilent Technologies, Santa Clara, CA, USA).

### 4.5. Parasites

The *P. falciparum* 3D7 strain was kept in RPMI 1640 medium with 25 mM NaHCO_3_, 25 mM HEPES (pH 7.4), 11 mM D-glucose, 3.67 mM hypoxanthine, and 50 mg/mL gentamicin, supplemented with 0.5% of the lipid-rich bovine serum albumin AlbuMAX II and incubated at 37 °C. The culture medium was changed daily. Parasitaemia was maintained below 10%, with 2.5% hematocrit in human O+ erythrocytes [33]. The Y strain of *T.cruzi* was isolated from a patient in the acute phase of Chagas disease [34], and the epimastigote forms were cultured at 28 °C in liver infusion tryptose (LIT) medium [35]. The cultures used had a maximum of six in vitro passages.

### 4.6. Animals

Healthy 4–6-year-old female BALB/c mice, purchased from the Institute of Science and Technology in Biomodels of Oswaldo Cruz Foundation, were used. Animal procedures were performed in accordance with the National Council for Control of Animal Experimentation (Conselho Nacional de Controle de Experimentação Animal—CONCEA) and approved by the local Ethics Committee on Animal Care and Utilization (CEUA-IOC L-018/2018).

### 4.7. Cell Culture

Hepatocarcinoma cells (HepG2) were cultivated in RPMI 1640 (Sigma, St. Louis, MI, USA) medium, and the *African green monkey* kidney VERO cell line was cultivated in Dulbecco’s modified eagle medium (DMEM) (Sigma, St. Louis, MI, USA). Cells were cultivated at 37 °C and 5% CO_2_, and the supplemented medium was changed every two days. Peritoneal macrophages were obtained from BALB/c mice elicited with 3 mL 3% thioglycolate for 72 h and kept in RPMI 1640 (Sigma, St. Louis, MI, USA) [36]. Both cells were supplemented with 10% fetal bovine serum, penicillin (100 U/mL), and streptomycin (100 μg/mL) at 37 °C and 5% CO_2_.

### 4.8. Antimicrobial Assay

The antimicrobial susceptibility was carried out by microbroth dilution assay [37]. Tests were performed on 96-well plates with 100 μL of Mueller Hinton Broth (MHB) (HiMedia, Mumbai, India), 100 μL of test compound, and 5 μL of test bacteria at 1.0 × 10^4^ CUF/mL, followed by incubation at 37 °C (24 h). The ravenelin obtained from the fungal culture was dissolved (initially 1mg) in 100 μL of dimethyl sulfoxide (DMSO) and 900 μL of brain heart infusion (BHI) broth given 1 mg/mL (3876 μM) stock solution. The stock solution was diluted from 3.876 μM to 0.95 μM for testing. *Escherichia coli* (ATCC 25922), *Pseudomonas aeruginosa* (ATCC 27853), *Salmonella typhimurium* (ATCC 14028), *Staphylococcus aureus* (ATCC 25923), and *Bacillus subtilis* (ATCC 6633) were provided by the Evandro Chagas Institute, Belém, Pará State, Brazil. Bioactivity was registered as red coloration absence in the wells after addition of 10 μL 2,3,5-triphenyltetrazolium chloride. The microorganisms were then sub-cultured on MHB plates. The activities of test compounds were classified as bacteriostatic or bactericidal according to the behavior of the microorganisms in these sub-cultures. Amoxicillin and terramycin were used as positive controls and the MHB culture medium was used as a negative control. The test was made in triplicate.

### 4.9. Antiplasmodial Assay

Parasites were synchronized to enrich the ring-stage parasites through sterile 5% (*m*/*v*) D-sorbitol treatment incubated at 37 °C for 10 min [38], followed by centrifugation at 600× *g* for 5 min. After resuspension of the centrifuged parasites, parasitemia was determined by microscope analysis of thin blood smears stained with Giemsa 10% solution after methanol fixation. Parasitemia was calculated from 1000 red blood cells (RBCs) with dilution of cultures of 0.5% parasitemia and 2% hematocrit by adding the appropriate volumes of erythrocytes and medium. A total of 180 µL of parasite aliquots were distributed into 96-well plates, previously prepared with 20 µL aliquots of a ten-fold concentrated compound. The controls were distributed in wells, with the negative and positive controls corresponding to non-parasitized erythrocytes, and parasite cultures in the absence of compounds were set in parallel. DMSO with a concentration below 0.05% (*v*/*v*) was used to assist in the solubilization of the compounds. Plates were organized in a humidified incubator with a gas mixture of 90% N_2_, 5% O_2_, and 5% CO_2_ at 37 °C for 72 h. After incubation, the culture medium was removed, followed by re-suspending in 100 µL phosphate-buffered saline (PBS) buffer (116 mM NaCl, 10 mM Na_2_HPO_4_, 3 mM KH_2_PO_4_) and lysing with 100 µL lysis buffer (20 mM Tris base, 5 mM EDTA, 0.0008% (*v*/*v*) Triton X-100, 0.008% (*m*/*v*) saponin, pH 8.0) containing 0.002% (*v*/*v*) SYBR Green I. Further incubation was performed at room temperature for 30 min, followed by determination of the parasitic density by fluorescence using a SpectraMAX Gemini EM plate reader (Molecular Devices Corp., Sunnyvale, CA, USA) (excitation at 485 nm, emission at 535 nm) [39]. Half maximal inhibitory concentration (IC_50_^Pf^) was determined by non-linear regression analysis of the resulting concentration–response curve using the software Origin 2016 (OriginLab Corporation).

### 4.10. Antitrypanosomal Activity Assay

The epimastigote forms of *T. cruzi* (2 × 10^6^ parasites/mL) from a 3–5-day-old culture were incubated for 24 h in 96-well plates in the presence of different concentrations of ravenelin (3.78–1000 μM) at least in triplicate, with a final volume of 100 µL per well. Wells without parasites were used as blank, and wells containing only parasites were used as control. The viability of parasites was evaluated after treatment by counting the total number of live epimastigotes, taking into account the flagellar motility, using a Neubauer chamber and light microscope. This count was compared with the score of non-treated epimastigote growth. This experiment was carried out in triplicate. The results are expressed as parasite growth inhibitory concentration (IC_50_). Benznidazole (3.0–384.3 μM), was used as reference drug. Activity against intracellular amastigotes was performed with peritoneal macrophages cultured in 24-well plates (5 × 10^5^ cells/well) with coverslips and infected with trypomastigotes forms of *T. cruzi* using a ratio of 10:1 parasite/cell. After 6 h, the cells were washed three times with PBS to remove free parasites. The infected cells were treated with ravenelin (0.242–3.87 μM) and benznidazole (24.0–384.3 μM) at least in triplicate for 24 h. Coverslips with the infected and treated cells were fixed with Bouin solution stained with Giemsa solution and examined under light microscopy. The intracellular amastigote number of one hundred cells was normalized and used to calculate the IC_50_ [40].

### 4.11. Cytotoxicity Test and Selectivity Index

Hepatocarcinoma cell and peritoneal macrophages were cultured in 96-well plates (5 × 10^5^ cells/mL) with different concentrations of ravenelin (7.5–242.0 μM) and benznidazole (24.0–768.5 μM) at least in triplicate up to a final volume of 100 μL per well at 37 °C and 5% CO_2_. Wells without cells were used as blanks, and wells with cells and DMSO 1% were only used as controls. After 24 h, cell viability was evaluated by modified colorimetric method with tetrazolium-dye 3-(4,5-dimethylthiazol-2-yl)-2,5-diphenyltetrazolium bromide (MTT) [41]. The results were used to calculate the 50% cell cytotoxicity (CC_50_). The selectivity index (SI) was obtained from the ratio of peritoneal macrophages CC_50_ and IC_50_ [42].

### 4.12. Statistical Analysis

The IC_50_ and CC_50_ were obtained from a nonlinear regression fit curve of concentration in log versus normalized response. The values were expressed as mean ± S.D. Analyses were performed using the software GraphPad Prism (Version 6.01, GraphPad Software Inc., La Jolla, San Diego, CA, USA), and differences were considered significant when *p* < 0.05.

## 5. Conclusions

The present work reported the isolation and structural characterization of ravenelin, a secondary metabolite from *E. rostratum*. Our findings indicated that the natural product is endowed with both antibacterial (*B. subtilis*) and antiparasitic (*P. falciparum* and *T. cruzi*) activities, with considerable selectivity indexes. These results suggest that ravenelin is an attractive candidate for hit-to-lead development, especially for Chagas Disease, a serious public health problem in Latin America.

## Figures and Tables

**Table 1:** Minimum inhibitory concentration (MIC) of ravenelin for Gram-negative and Gram-positive bacteria.

Compound	MIC (µM)				
	Gram (+) Bacteria		Gram (−) Bacteria		
	*B. subtilis*	*S. aureus*	*E. coli*	*P. aeruginosa*	*S. typhimurium*
Ravenelin	7.5	484	>1000	>1000	>1000
Amoxicillin	1.3	21.4	21.4	21.4	21.4
Terramycin	16.3	16.3	16.3	16.3	16.3

MIC: minimum inhibitory concentration. *Bacillus subtilis* (ATCC 6633), *Staphylococcus aureus* (ATCC 25923), *Escherichia coli* (ATCC 25922), *Pseudomonas aeruginosa* (ATCC 27853), and *Salmonella typhimurium* (ATCC 14028).

**Table 2:** Antiprotozoal activities of ravenelin.

Compounds	*P. falciparum*	*T. cruzi*	
	IC_50_ (µM)	Epimastigote	Intracellular Amastigote
		IC_50_ (µM)	IC_50_ (µM)
Ravenelin	3.4 ± 0.4	5 ± 1	9 ± 2
Artesunate	0.0085 ± 0.0008	NA	NA
Benznidazole	NA	22 ± 1	2 ± 1

Data represents mean ± SD of at least two experiments. IC_50_: inhibitory concentration for 50% of parasite inhibition. NA, not applicable.

**Table 3:** Cytotoxicity in HepG2 cell and BALB/c peritoneal macrophages, and selectivity index of ravenelin.

Compound	HepG2	SI ^a^	Peritoneal Macrophage	SI ^b^
	CC_50_ (µM)		CC_50_ (µM)	
Ravenelin	>50	>15	185 ± 1	21
Artesunate	110 ± 30	13,000	NA	NA
Benznidazole	NA	ND	666 ± 1	333

Data represents mean ± SD of at least two experiments realized in triplicate. CC_50_: cytotoxic concentration for 50% of cells; SI ^a^: selectivity index in relation to inhibitory concentration for 50% of *P. falciparum* 3D7. SI ^b^: selectivity index in relation to inhibitory concentration for 50% of *T. cruzi* intracellular amastigote. NA, not applicable; ND, not determined.

## Data Availability

The data presented in this study are available in Supplementary Material.

## Supplementary Materials

The following are available online, Figure S1–S7: NMR, IR, and MS spectra of the active compound are available online.

## Sample Availability

Samples of the compounds ravenelin are available from the authors.
