# Supplementary material for: Antiprotozoal and Antibacterial Activity of Ravenelin, a Xanthone Isolated from the Endophytic Fungus Exserohilum rostratum
Source: Molecules. 2021 Jun 2;26(11):3339. doi: 10.3390/molecules26113339 (PMC8199546; doi:10.3390/molecules26113339)
Supplement: Supplementary file 1 [file molecules-26-03339-s001.zip › molecules-1195254-supplementary.pdf]

Supplementary Information

Antiprotozoal and Antibacterial Activity of Ravenelin, a Xanthone Isolated from the Endophytic Fungus *Exserohilum rostratum*

Jeferson Rodrigo Souza Pina [1], João Victor Silva-Silva [2], Josiwander Miranda Carvalho [1], Heriberto Rodrigues Bitencourt [1], Luciano Almeida Watanabe [1], Juan Matheus Pereira Fernandes [2], Guilherme Eduardo de Souza [3], Anna Caroline Campos Aguiar [3], Rafael Victorio Carvalho Guido [3], Fernando Almeida-Souza [2,4], Kátia da Silva Calabrese [2], Patrícia Santana Barbosa Marinho [1] and Andrey Moacir do Rosario Marinho [1,*]

[1] Post-Graduate Program in Chemistry, Federal University of Pará, 66075-110, Belém, Brazil; konanquim@gmail.com (J.R.S.P.); mcwander@hotmail.com (J.M.C.); heriberto.ufpa@gmail.com (H.R.B.); lucianowat@yahoo.com.br (L.A.W.); pat@ufpa.br (P.S.B.M.)

[2] Laboratory of Immunomodulation and Protozoology, Oswaldo Cruz Institute, Oswaldo Cruz Foundation, 21040-360 Rio de Janeiro, RJ, Brazil; jvssilva89@gmail.com (J.V.S.-S.); juanfernandes222@gmail.com (J.M.P.F.); fernandoalsouza@gmail.com (F.A.-S.); kscalabrese@gmail.com (K.d.S.C.)

[3] São Carlos Institute of Physics, University of São Paulo, São Carlos, 13566-590, São Paulo, Brazil; guilherme.eduardo.souza@usp.br (G.E.d.S.); carolcaguiar@yahoo.com.br (A.C.C.A.); rvcguido@ifsc.usp.br (R.V.C.G.)

[4] *Post-Graduate Program Animal Sciences*, State University of Maranhão, São Luís, 65055-310 Maranhão, Brazil

*Correspondence: andrey@ufpa.br; Tel.: +55-91-3201-8050

Figure S1. NMR ^{1}H spectrum to ravenelin (**1**) (400 MHz, C_3D_6O)

Figure S2. NMR ^{13}C spectrum to ravenelin (**1**) (100 MHz, C_3D_6O)

Figure S3. HMBC spectrum to ravenelin (**1**)

Figure S4. HSQC spectrum to ravenelin (**1**)

Figure S5. COSY spectrum to ravenelin (**1**)

Figure S6. IR spectrum to ravenelin (**1**)

Figure S7. ESIMS(-) spectrum to ravenelin (**1**)

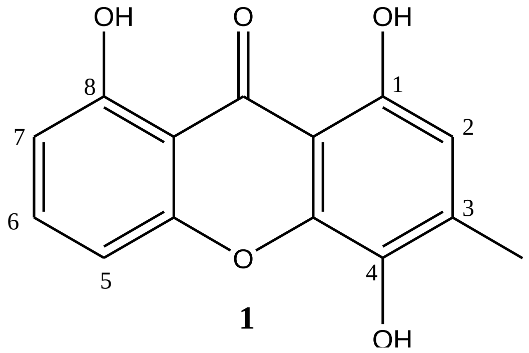

1

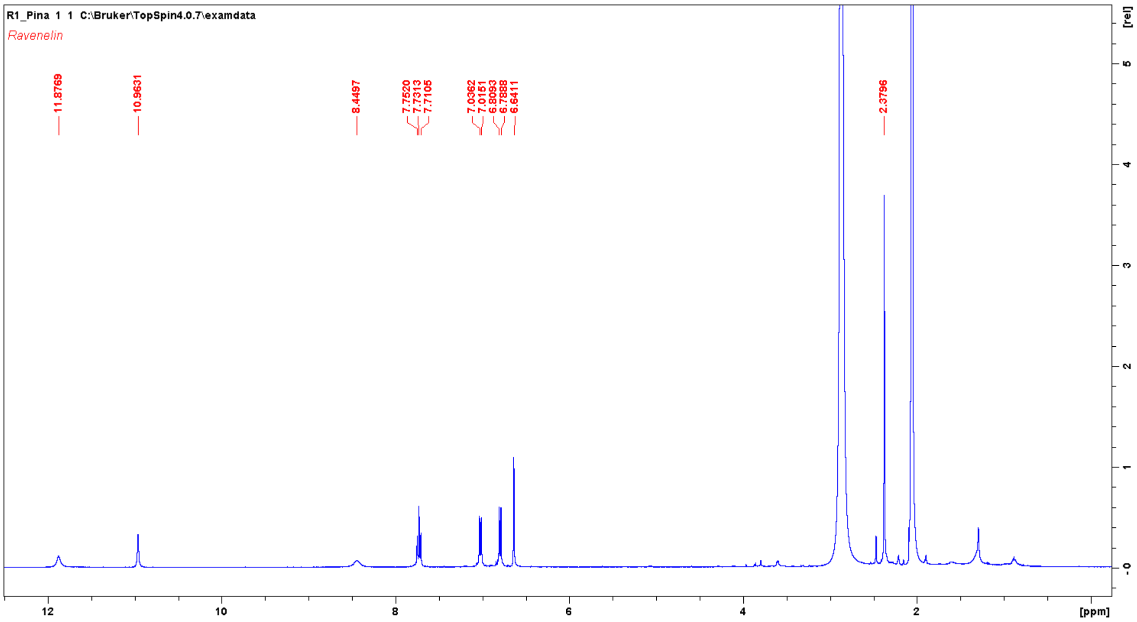

Figure S1. NMR ^{1}H spectrum to ravenelin (**1**) (400 MHz, C_3D_6O)

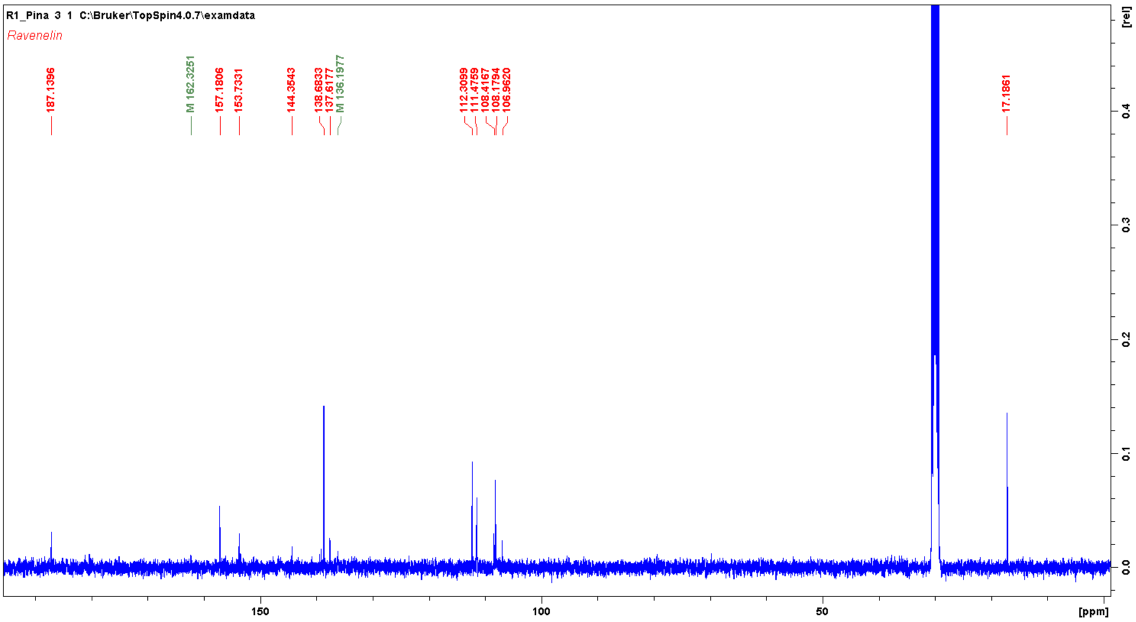

Figure S2. NMR ^{13}C spectrum to ravenelin (**1**) (100 MHz, C_3D_6O)

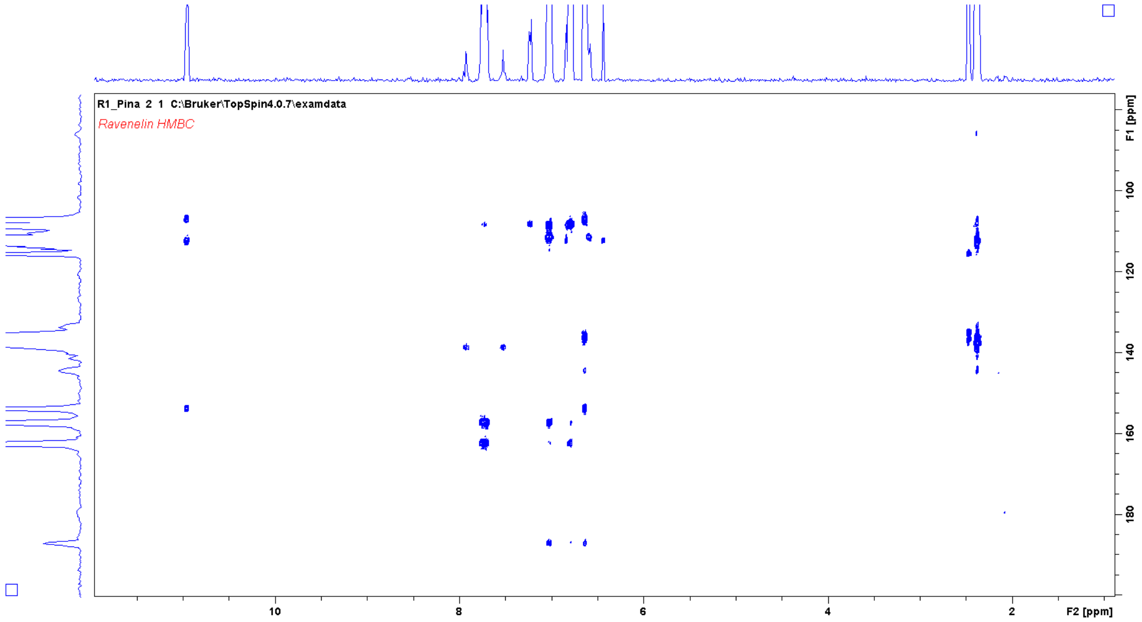

Figure S3. HMBC spectrum to ravenelin (**1**)

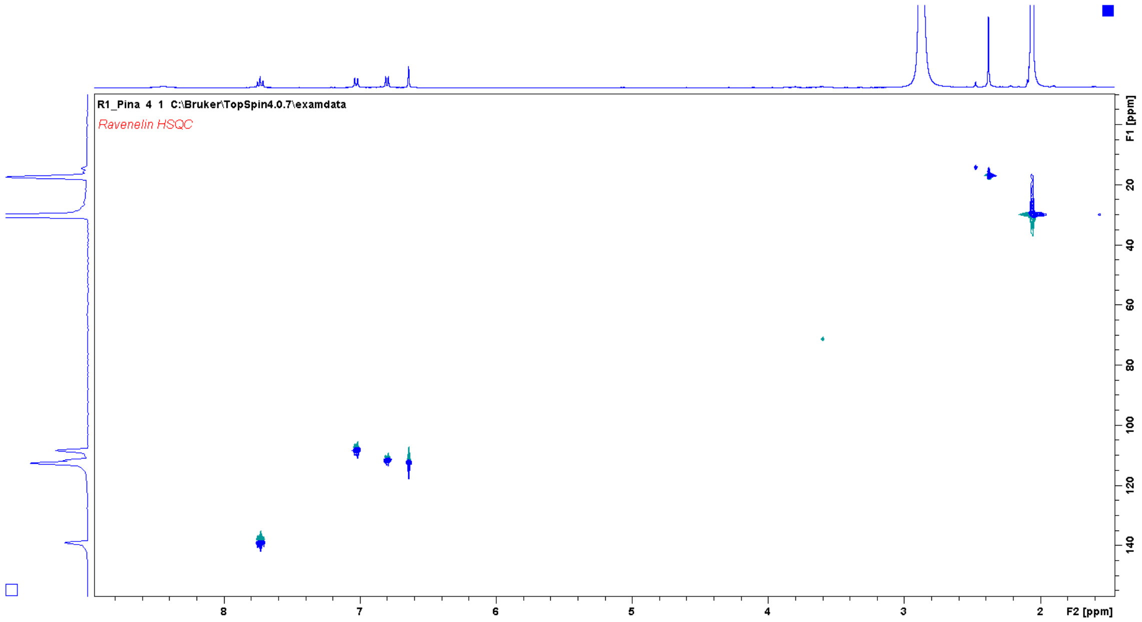

Figure S4. HSQC spectrum to ravenelin (**1**)

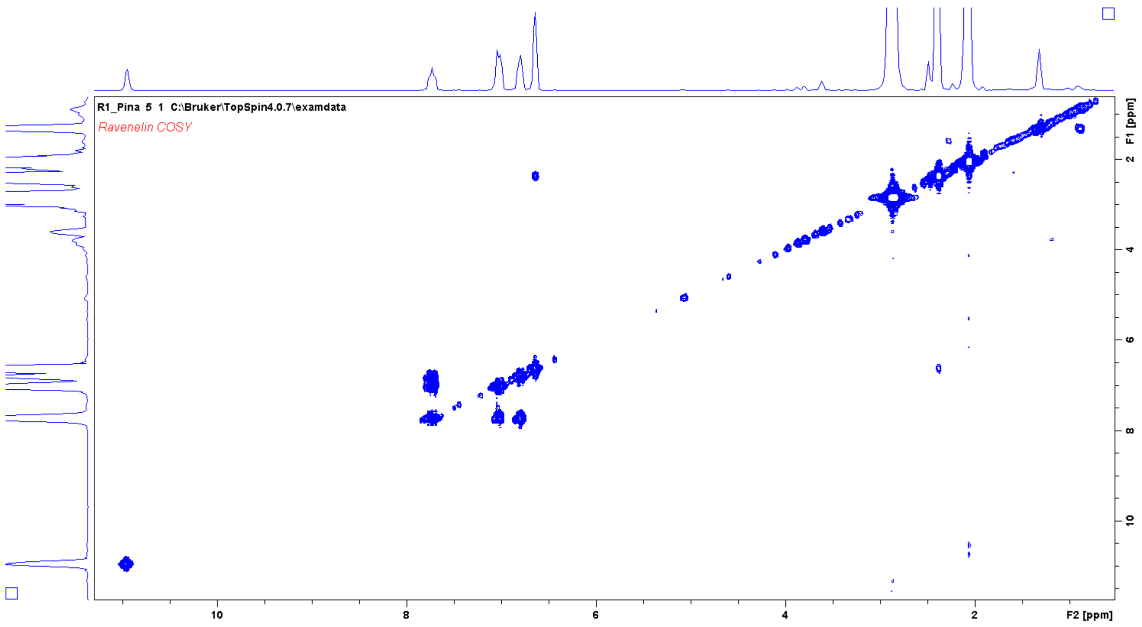

Figure S5. COSY spectrum to ravenelin (**1**)

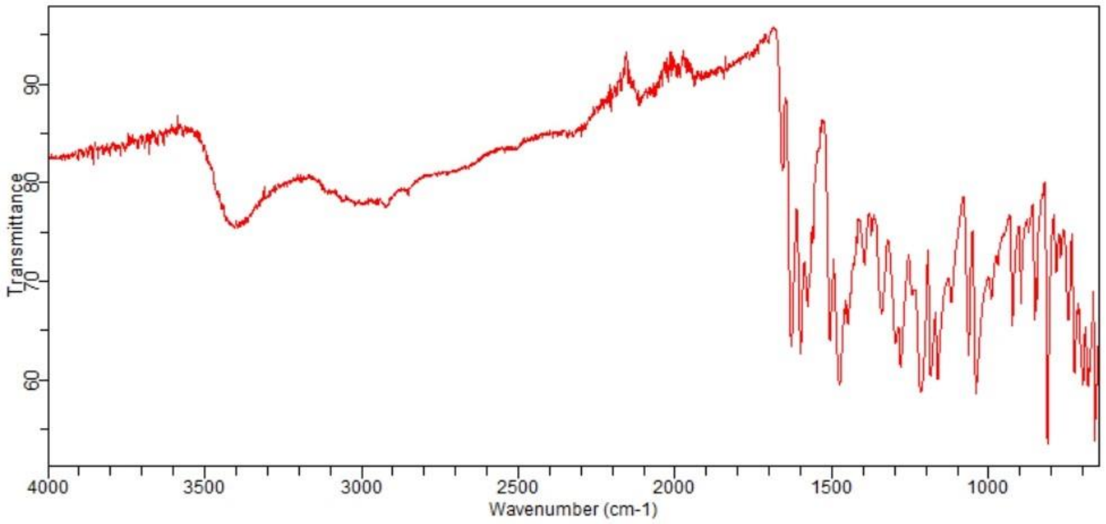

Figure S6. IR spectrum to ravenelin (**1**)

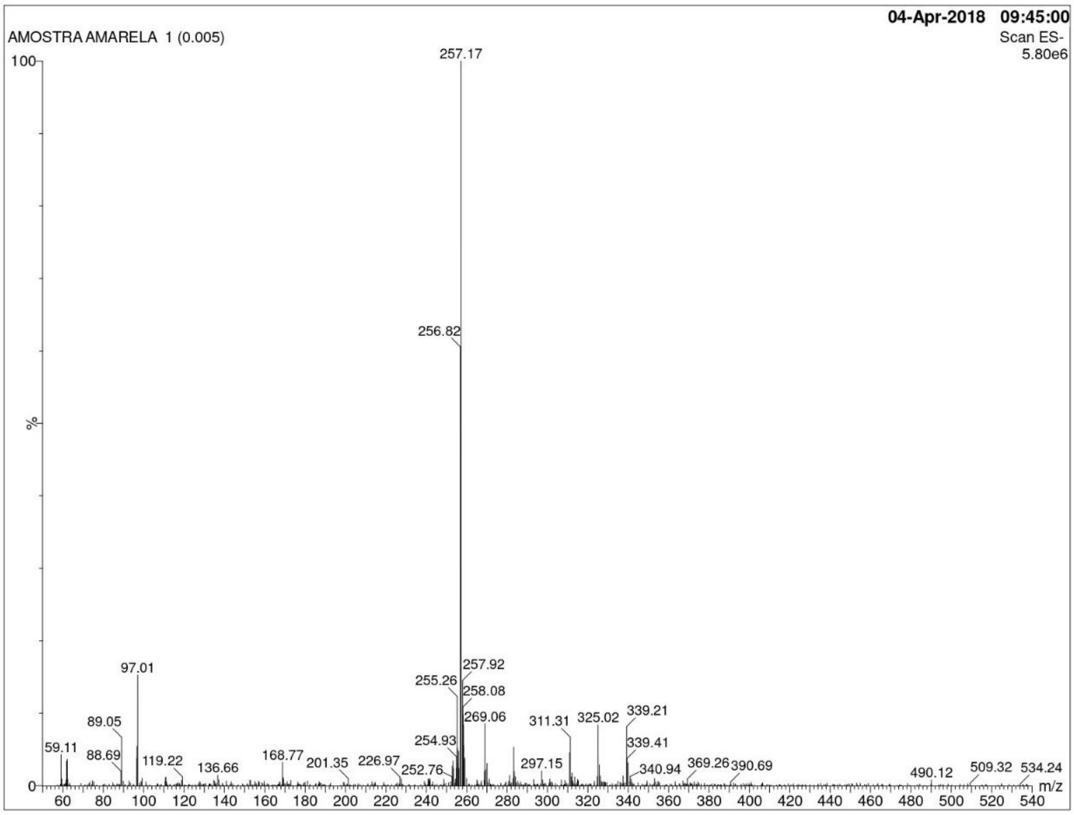

Figure S7. ESIMS(-) spectrum to ravenelin (**1**)
